# Supplementary material for: Antimicrobial susceptibility profile of Gonococcal isolates obtained from men presenting with urethral discharge in Addis Ababa, Ethiopia: Implications for national syndromic treatment guideline
Source: PLoS One. 2020 Jun 1;15(6):e0233753. doi: 10.1371/journal.pone.0233753 (PMC7263590; doi:10.1371/journal.pone.0233753)
Supplement: Attachment — Submitted filename: Repsonse to reviewers.docx [file pone.0233753.s002.docx]

1. When submitting your revision, we need you to address these additional requirements.

Please ensure that your manuscript meets PLOS ONE's style requirements, including those for file naming. The PLOS ONE style templates can be found at

<http://www.journals.plos.org/plosone/s/file?id=wjVg/PLOSOne_formatting_sample_main_body.pdf> and <http://www.journals.plos.org/plosone/s/file?id=ba62/PLOSOne_formatting_sample_title_authors_affiliations.pdf>

2. Please ensure that you include a title page within your main document. You should list all authors and all affiliations as per our author instructions and clearly indicate the corresponding author.

Additional Editor Comments (if provided):

Please revise this manuscript addressing in full all the comments and suggestions the reviewers have provided. In particular pay attention to providing all the data, and ensuring that all figures and tables are properly presented, annotated and referenced and discussed in the text. It is recommended that you seek editorial assistance to ensure the manuscript is written in grammatically correct, intelligible, standard English.


[Note: HTML markup is below. Please do not edit.]

Reviewers' comments:

Reviewer's Responses to Questions

**Comments to the Author**

1. Is the manuscript technically sound, and do the data support the conclusions?

The manuscript must describe a technically sound piece of scientific research with data that supports the conclusions. Experiments must have been conducted rigorously, with appropriate controls, replication, and sample sizes. The conclusions must be drawn appropriately based on the data presented.

Reviewer #1: Yes

Reviewer #2: Partly
**Response to reviewer**

**Dear reviewers, thank you for the constructive comments. We have tried to address the comments accordingly.**

2. Has the statistical analysis been performed appropriately and rigorously?

Reviewer #1: Yes

Reviewer #2: Yes

**Dear reviewers, thank you.**

3. Have the authors made all data underlying the findings in their manuscript fully available?

The [PLOS Data policy](http://www.plosone.org/static/policies.action#sharing) requires authors to make all data underlying the findings described in their manuscript fully available without restriction, with rare exception (please refer to the Data Availability Statement in the manuscript PDF file). The data should be provided as part of the manuscript or its supporting information, or deposited to a public repository. For example, in addition to summary statistics, the data points behind means, medians and variance measures should be available. If there are restrictions on publicly sharing data—e.g. participant privacy or use of data from a third party—those must be specified.

Reviewer #1: No

Reviewer #2: No

**Response to reviewers:**

**Dear reviewers: We cannot share all data publicly in the present study because of the restriction related to participant privacy.**

4. Is the manuscript presented in an intelligible fashion and written in standard English?

PLOS ONE does not copyedit accepted manuscripts, so the language in submitted articles must be clear, correct, and unambiguous. Any typographical or grammatical errors should be corrected at revision, so please note any specific errors here.

Reviewer #1: No

Reviewer #2: No

Response to reviewers:

**Authors would like to thank you for your constructive comments. We have tried to fix typos and grammatical errors. Thank you!**

5. Review Comments to the Author

Please use the space provided to explain your answers to the questions above. You may also include additional comments for the author, including concerns about dual publication, research ethics, or publication ethics. (Please upload your review as an attachment if it exceeds 20,000 characters)

Reviewer #1: The manuscript describes a study of urethral discharges from male patients in Addis Ababa, Ethiopia. It is motivated by the growing problem of antibiotic resistance in Neisseria Gonorrhoea. Its main findings are that gonococcal isolates are detected in 69% of those discharges. Of these isolates, a significant proportion were non-susceptible to fluoroquinolone, which is part of the basis of gonorrhoea management in sub-Saharan African countries including Ethiopia. The scientific work is sound but considerable improvement is required in its presentation.

1) The manuscript appears to be technically sound. The study is described with an appropriate level of detail and its design was clearly suitable for the topic of interest so that one would expect its data to be capable of supporting its conclusions. However the actual data are not supplied.
2) The statistical analysis is appropriate to the question at hand and done correctly.
3) The authors have not made their data available. It is not in the manuscript, nor is there a link provided, and there is no supplementary section.
4) The English in this manuscript does not meet the standards expected for a credible, reviewed scientific journal. It is intelligible most of the time but replete with grammatical errors and poorly worded phrases, too many to outline here. An exception is paragraph five in "Results and Discussion" in which figure one is referenced and discussed. The authors presumably mean to say that ceftriaxone non-susceptibility was not found in any isolates. The rest of this paragraph is incomprehensible. The authors are advised to rewrite the manuscript with assistance from a native speaker of English.

**Response to reviewer:**

**Authors would appreciate the positive words. We are not able to share all the data due to participant privacy. The grammatical problems have been reviewed and fixed as per the comments by the reviewer. Thank you.**


Additional Comments:
A) The presentation of percentages throughout the manuscript is inconsistent, most notably in the third paragraph of the "Results and Discussion" section and in table 1. It is indicated that percentages are being presented but some figures have "%" signs while others do not. The "%" should be used in text while the convention used in table 3 should be used consistently in all tables.

**Response to reviewer:**

**Thank you for the valuable comments. All the comments have been addressed accordingly.**

b) Table 2 is not referenced in the text although it is apparently relevant to the fourth paragraph in "Results and Discussion". Also, most of its data appears to be missing which renders the table confusing. As far as I can make out, each row should add up to 100 (%) but they do not. This table should be either corrected and properly referenced, or deleted.

**Response to reviewer:**

**Thank you for the valuable comments. All the comments have been addressed accordingly.**

c) The page with table 2 has what appears to be a footnote entitled "MIC Ranges for Resistance and Intermediate Susceptibility as defined by CLSI Guidelines". This useful and relevant information should be clearly presented in its own table and appropriately referenced from within the text.

**Response to reviewer:**

**Thank you for the valuable comments. All the comments have been addressed accordingly.**

d) The bars in table 1 have no apparent order. They should be plotted in order and positioned to scale on the x-axis since these categories are defined by the quantifiably measurable MIC.

**Response to reviewer:**

**Thank you for the valuable comments. All the comments have been addressed accordingly.**

e) It is stated in the final paragraph of the "Results and Discussion" section and again in the "Conclusions" that this work has already led to a revision of Ethiopia's national guidelines for managing patients with urethral discharge, but no description or reference to a description is given. The revisions should be described and ideally a paper or relevant government document should be referenced.

**Response to reviewer:**

**Thank you for the valuable comments. All the comments have been addressed accordingly. The revised document has been referenced.**


f) References to tables and figures should use a capital letter, "Table 3" instead of "table 3". This was sometimes neglected, particularly for Table 3 and Figure 1.

**Response to reviewer:**

**Thank you for the valuable comments. All the comments have been addressed accordingly.**

Reviewer #2: Antimicrobial-resistant gonorrhea is a major public health threat. Therefore, manuscript addressing the antimicrobial susceptibility of Neisseria gonorrhoeae (NG) isolates are important. However, this manuscript, as written, lacks the necessary level of detail and scientific rigor to be published in PLOS one Medicine.

**Response to reviewer:**

**Authors greatly appreciate reviewer’s constructive comments and criticism. We have tried to revise the manuscript as per the comment given.**

Major issues.

Introduction.

1) Does not describe the current WHO recommended treatment for gonorrhea. This is important in order to provide context for the recommended treatment in Ethiopia.

2) There are several recent papers describing the susceptibility of NG isolates in Africa which have found that most NG isolates from African countries are resistant to Ciprofloxacin.

3) The references cited are older. A quick search on Pubmed could yield a list of recent publications on this topic.

4) Overall, the introduction is too short.

**Response to reviewer:**

**Dear reviewer, thank you for all the comments. The introduction section has been revised accordingly.**

Results/discussion

1) Although table 1 describes the gram stain results, a discussion of gram stain results in comparison to culture results should be included.

**Response to reviewer:**

**Thank you for this very important comment, we have attempted to include this.**

2) The authors need to be careful in attributing the high level of NG infections to the fact that the samples were collected from men. The reason for the high positivity rates is because the samples were collected from symptomatic subjects.

**Response to reviewer:**

**Comment very valid and therefore has been considered, thank you.**

3) The discussion on the implication for National syndromic treatment guideline need to be expanded. It is currently limited to one line. “Antimicrobial Susceptibility Profile of Gonococcal Isolates Obtained from Men Presenting with Urethral Discharge: Implication for National Syndromic Treatment guideline”

**Response to reviewer:**

**Thank you for this vital comment, it has been addressed per the comment.**

The title should also include Ethiopia to better guide the reader.

**Response to reviewer:**

**The word “Ethiopia” has been included as per the comment, thank you.**

Overall, this manuscript is suitable for a journal like STD which is more related to public health

**Response to reviewer:**

**We thank the reviewer for the vital comments.**
