# Supplementary material for: Antimicrobial susceptibility profile of Gonococcal isolates obtained from men presenting with urethral discharge in Addis Ababa, Ethiopia: Implications for national syndromic treatment guideline
Source: PLoS One. 2020 Jun 1;15(6):e0233753. doi: 10.1371/journal.pone.0233753 (PMC7263590; doi:10.1371/journal.pone.0233753)
Supplement: Attachment — Submitted filename: Response to reviewers May 11 2020.docx [file pone.0233753.s004.docx]

The authors have, on the whole, addressed the concerns expressed by the reviewers and the manuscript is much improved. There remain a couple of minor issues that need to be addressed before I can recommend this manuscript for publication;

**Response to the Editor:**

**Dear editor, thank you so much for all the constructive comments. And thank you also for this last opportunity, again, for us to be able to publish our finding. We have tried to address the major issue raised accordingly.**

1) the frequency columns in tables 1-3 are redundant and should be removed because the identical information in provided in the 'N (%)' column

**Response to the Editor:**

**The correction has been made. The frequency columns in all tables has been removed. Thank you so much for due diligence.**

2) the total for Gram stain is missing in Table 2

**Response to the Editor**

**The correction has been made. The total for gram stain has been added. Thank you so much for due diligence.**

3) Table 4 does not contain information regarding multi-drug non-susceptibility as stated on page 9. This information needs to be added to the table or presented in a separate table.

**Response to the Editor**:

**Correction has been made to the proportion of isolates with non-susceptibility to ciprofloxacin and pencillin and ciprofloxacilin and spectinomycin (In combination) and the reference made to table-4 was removed because it is presented in a narrative form in page 9. In the previous submissions, we added the information in the bottom 2 rows of table -4 and later omitted it because that has caused a confusion to the reviewers as the numbers in these rows did not add up to hundred. Since we only had the combined non-susceptibility for the two agents so did not feel to put the information in a table of its own rather we have indicated in the narrative on page 9.**

4) The results presented in Figure 1 are not adequately described in the results on page 9 and the cut-off MIC is not specified so this cannot be interpreted. There is also no figure legend for Figure 1, only a figure title.

**Response to the Editor:**

**Thank you for the comment. We have attempted to describe the result presented in the figure on page 9 and also have included figure legend as commented on page 9 right immediately after the paragraph where the figure was referenced.**
